# Supplementary material for: Proteomic Analysis of Maternal Urine for the Early Detection of Preeclampsia and Fetal Growth Restriction
Source: J Clin Med. 2021 Oct 13;10(20):4679. doi: 10.3390/jcm10204679 (PMC8537852; doi:10.3390/jcm10204679)
Supplement: Supplementary file 1 [file jcm-10-04679-s001.zip › jcm-1397664-supplementary.pdf]

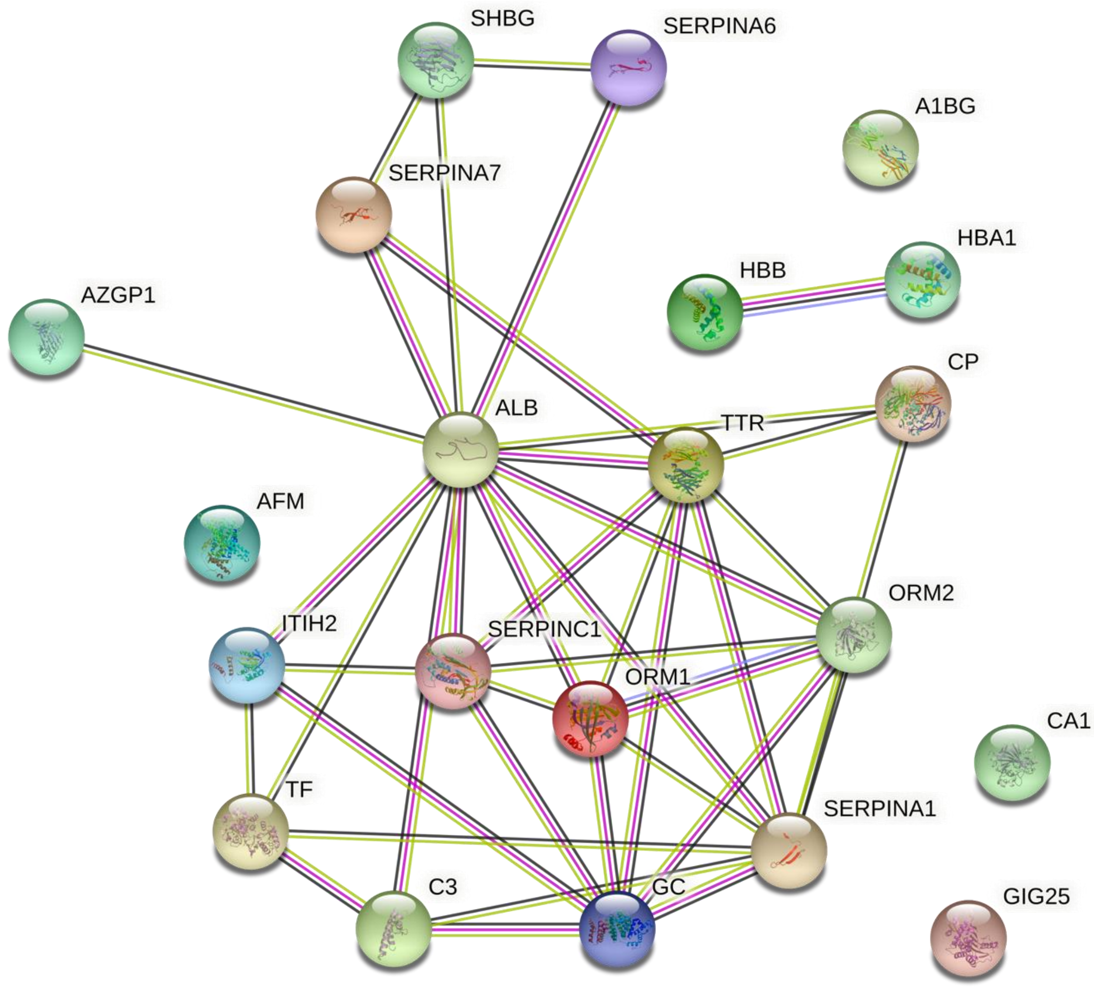

Supplementary Figure S1 STRING interaction network of the 21 proteins selected for validation study. Only interactions with high confidence interaction scores (> 0.7) are shown. Know protein interaction (experimentally demonstrated) (*pink*), gene co-expression (*black*), textmining (cited together in PubMed abstracts) (yellow), protein homology (*light purple*).
